# Cross-Sectional Study of Bone Mineral Density in Chronic Stroke According to Walking Speed

**DOI:** 10.3390/jcm14238426

**Published:** 2025-11-27

**Authors:** Maria-Arantzazu Ruescas-Nicolau, M. Luz Sánchez-Sánchez, Mónica Ahulló, Carmen Ballester-Estevan, Marco Iosa

**Affiliations:** 1 Physiotherapy in Motion, Multispeciality Research Group (PTinMOTION), Department of Physiotherapy, Faculty of Physiotherapy, University of Valencia, Gascó Oliag n 5, 46010 Valencia, Spain; arancha.ruescas@uv.es; 2 INCLIVA Biomedical Research Institute, 46010 Valencia, Spain; mahullo@incliva.es; 3 Department of Physiotherapy, Faculty of Physiotherapy, University of Valencia, Gascó Oliag n 5, 46010 Valencia, Spain; baescar@alumni.uv.es; 4 Department of Psychology, Sapienza University of Rome, Via dei Marsi 78, 00185 Rome, Italy; marco.iosa@uniroma1.it; 5 Smart Laboratory, Santa Lucia Foundation IRCCS, Via Ardeatina 306, 00179 Rome, Italy

**Keywords:** chronic stroke, walking speed, bone quality, bone mineral density, sound of speed, broadband ultrasonic attenuation, T-score, Z-score

## Abstract

**Background/Objectives**: Bone mineral density (BMD) assessments are uncommon in people with chronic stroke, and the relationship between BMD and gait speed remains poorly understood. This study examined between-limb differences in BMD of individuals with chronic stroke and limited versus non-limited community ambulation and analyzed the relationship between BMD and gait speed. **Methods**: This cross-sectional study included people with chronic stroke divided into two groups by walking speed (slow group [SG], <0.8 m/s, n = 38, and fast group [FG], ≥0.8 m/s, n = 46) and age- and sex-matched healthy individuals (control group [CG], n = 35). All participants underwent calcaneal ultrasound densitometry. **Results:** All the BMD parameters differed significantly between limbs in the SG, with the affected side demonstrating inferior outcomes. The FG only exhibited a significant difference in the broadband ultrasonic attenuation, with lower values on the affected side. Among groups, the SG demonstrated lower values in the affected leg for all parameters compared with the corresponding limb of both the FG and the CG. Furthermore, the SG demonstrated reduced speed of sound (SOS) in the non-affected limb compared with the FG’s in theirs. Multiple regression analysis revealed that the ambulation ability, the affected gastrocnemius spasticity, disability, and SOS of the affected limb together explained 71.9% of the gait speed variance. **Conclusions**: Among stroke survivors, a slower gait speed is associated with greater between-limb differences in BMD. SOS in the affected limb emerged as a key predictor of gait speed. This highlights the need for more thorough BMD evaluations for stroke patients.

## 1. Introduction

Stroke is a leading cause of disability worldwide [1]. At the turn of the century, the incidence of stroke in Europe ranged from 95 to 290 cases per 100,000 inhabitants per year [2]. However, the number of people living with post-stroke sequelae in the European Union is estimated to increase by 27% between 2017 and 2047, mainly due to an aging population and the improved survival rates [3].

The International Classification of Functioning, Disability and Health categorizes post-stroke sequelae into the following dimensions: body function and structure, activity, and participation [4]. Among other structural and bodily function deficits, people with stroke sequelae have reduced bone mineral density (BMD) [5]. Physical inactivity, malnutrition, hemiparesis, and reduced sun exposure are factors that contribute to the relationship between stroke and reduced bone mineral content [5]. In fact, there is evidence that stroke survivors are at increased risk of developing osteoporosis [6]. Bone loss begins immediately after the stroke, continues for up to three to four months after onset, and progresses at a slower rate for up to a year after onset [7]. Furthermore, ongoing trabecular bone loss has been observed in the distal epiphysis of the affected tibia in people with chronic stroke, and it tends to stabilize two years after the event [8]. Interestingly, longitudinal studies have shown that stroke severity may influence the rate of change and timing of the plateau phase of BMD [9]. In this regard, it has been observed that the decline in hip BMD one year after a stroke varies according to an individual’s functional status; that is, people who remained wheelchair-bound experienced a much greater decline in hip BMD (13%) than those who regained ambulatory function two months after a stroke (8%) or who were ambulatory at stroke onset (3%) [10]. This highlights the importance of achieving an adequate mobility status following a stroke.

Although the recovery of walking capacity is one of the main aims in stroke rehabilitation, many stroke survivors in the chronic phase continue to have limited ambulatory capacity, including reduced gait speed [11]. Gait speed is a widely used measure of mobility and an important low-cost clinical indicator of physical function, as it is assessed by the time taken to comfortably walk a predetermined distance. Gait speed is regarded as a reliable indicator of recovery in walking ability [12]. Therefore, failing to reach a certain gait speed could potentially cause problems in daily life and limit participation in social events, such as keeping up with a partner or crossing the street [13]. Reduced gait speed is also associated with loss of independence [14]. All these aspects could ultimately result in individuals abstaining from walking and participating in physical activities, which would increase their risk of mortality [15]. Furthermore, gait speed is a key predictor of quality of life in stroke survivors [16]. Along with walking distance, gait speed is the dominant predictor of walking ability at home or in the community [12]; that is, it enables people to be categorized based on their walking ability. In that sense, the cut-off of 0.8 m/s is used to discriminate between limited and unlimited community ambulation [17].

Evidence also suggests that habitual walking speed is a significant predictor of self-reported functional capacity in people with chronic stroke, accounting for 48% of its variance [18]. It is common for stroke survivors to have reduced functional capacity [19]. Interestingly, lower functional capacity has been consistently associated with low BMD in diverse populations, including children and adolescents with reduced mobility [20], people with ankylosing spondylitis [21], and the elderly [22]. However, a recent meta-analysis claimed that the impact of functional capacity on bone health in stroke patients remains understudied [9], and although stroke is a chronic condition, few people with stroke are currently screened for bone loss, and even fewer are treated [23]. This underscores the importance of studying bone properties in this population. This is particularly salient given that the inability to walk independently is associated with lower BMD [24]. However, to the best of our knowledge, no studies have been conducted to determine whether self-selected walking speed can influence bone health. Specifically, it is unclear whether people with stroke sequelae whose walking speed limits their mobility in the community have poorer BMD in their lower limbs than those whose walking speed does not limit their mobility. This information could help identify people who require an in-depth bone study at the clinic, encouraging the clinical screening of individuals at risk or in need of such a study [17].

Therefore, the objective of this study was to analyze between-limb differences in BMD in individuals with chronic stroke and limited versus non-limited community ambulation and compared with healthy controls. Moreover, the aim was to analyze the relationship between BMD and walking speed in chronic stroke. It was hypothesized that subjects in the chronic phase post-stroke who demonstrate full community ambulation speed would have lower differences in BMD parameters between affected and non-affected limbs than their counterparts with limited community ambulation. In addition, we hypothesized that some of the BMD variables studied would be related to the walking speed in chronic stroke.

## 2. Materials and Methods

### 2.1. Study Design and Participants

A cross-sectional study was conducted between December 2022 and July 2024 at the Faculty of Physiotherapy of the University of Valencia (Valencia, Spain) in subjects with chronic stroke (stroke group) and healthy counterparts (control group [CG]) matched by age and sex. Participants of the stroke group were divided based on gait speed in slow group (SG; speed < 0.8 m/s) and fast group (FG; speed ≥ 0.8 m/s, able to ambulate at full community speed) [17].

Adults (age ≥ 18 years) with motor deficits due to chronic stroke (onset ≥ six months) were recruited from brain injury associations in the region of Valencia (Spain), while healthy participants were recruited through personal contact and from the authors’ institution. Stroke participants were included if they lived in the community, could walk ten meters indoors with or without an assistive device and without supervision (Functional Ambulation Classification of the Hospital of Sagunto [FACHS] ≥ 2) [25], and had sufficient cognitive capacity (assessed by a neuropsychologist expert in brain injury) to provide informed consent and understand verbal instructions to undergo the assessment tests. In addition, the following exclusion criteria were applied to all the participants: (1) presence of any condition that affects bone metabolism (e.g., chronic renal, hepatic, or thyroid disease, or prolonged treatment with corticosteroids, anticonvulsants, or thyroid hormones); (2) use of prescribed bone-specific medications (i.e., bisphosphonates, hormone replacement therapy, calcitonin, and vitamin D); (3) having pain (visual analog scale ≥ 3); (4) suffering from any medical condition or disorder that could interfere with the assessment tests (e.g., blindness or severe alteration of sensation); and (5) having severe musculoskeletal (e.g., recent surgery or amputations) or neurological conditions other than stroke (e.g., Parkinson’s disease).

A prior sample size estimation was performed using G*Power v3.1 software (University of Dusseldorf, Dusseldorf, Germany). For this purpose, data from a previous study about the relationship between BMD and stroke-related motor impairment were used [24]. Specifically, the sample size was calculated based on the mean Z-score difference in the affected hip between people with chronic stroke who had and did not have limited community ambulation. In order to achieve the study’s primary objective, a sample size of 96 participants (32 per group) was determined based on a power of 80%, an alpha error of 95%, and an expected small effect size (f = 0.12) [24]. Finally, to ensure the study’s maximum power, 131 subjects were recruited using consecutive sampling until the study’s design reached time and date saturation.

The protocol and objectives of the study were explained to the participants prior to enrollment, and written informed consent was obtained before participation. The study protocol was approved by the Human Research Ethics Committee of the University of Valencia, Spain (register number 2308291), and the study was conducted in accordance with the tenets of the Declaration of Helsinki. This article conforms to the STROBE guidelines (see Supplementary Materials).

### 2.2. Procedures

Each participant was assessed in a single session. They were instructed to wear comfortable clothing and their usual walking shoes for the assessment. All clinical measures were administered by trained personnel.

Initially, sociodemographic (age, sex, and ethnicity), clinical data (smoking and alcohol consumption habits, medication, use of mobility aids, and stroke history, that is, number of events, side affected, time since stroke, and type), and the number of falls in the preceding year were collected from medical records and via clinical interview. A fall was defined as an involuntary event in which the body loses balance and hits the ground or another solid surface that stops it [26]. In healthy individuals, leg dominance was determined by asking the question, “If you had to kick a ball towards a target, which leg would you use?” [27]. Subsequently, clinical and functional measures were performed.

### 2.3. Measures

The Montreal Cognitive Assessment (MoCA, score range 0–30) was used to evaluate cognitive status [28], with lower scores indicating more severe cognitive decline. The MOCA has shown high degree of sensitivity (90%) in differentiating cognitive impairment from non-cognitive impairment in chronic stroke [29], and it has exhibited high test–retest reliability (ICC = 0.85) in people with stroke [30].

The Functional Ambulation Classification of Hospital of Sagunto (FACHS) was subsequently utilized to determine walking ability [25]. This classification ranges from 0 to 5, with higher scores denoting better ambulation capacity. This scale has demonstrated good inter-rater reliability (Kappa coefficient = 0.74) and strong construct validity, as supported by significant linear correlations between FAC scores and both walking velocity (Spearman’s rho = 0.84), and the number of steps taken (Spearman’s rho = 0.86) [25].

Next, the modified Rankin scale (mRS, score range 0–6) was used to assess functional independence [31], with higher scores indicating higher disability. Research of its psychometric properties in the context of stroke has shown that this scale exhibits adequate validity when evaluated against stroke-related measures and demonstrates acceptable inter-rater reliability (Kappa coefficient = 0.55) [32].

To proceed with the evaluation, the subjects’ height and weight were measured with a precision of 0.1 cm and 0.1 kg, respectively, and body mass index (BMI; kg/m^2^) was calculated. This was followed by the bilateral assessment of the tibialis anterior and gastrocnemius muscle tone by using the modified Ashworth scale (MAS) [33]. The MAS has been proposed to be indicative of muscle hypertonia, given its relationship with alpha motoneuron excitability [34]. The reliability of the MAS for assessing increased muscle tone post-stroke in lower limb muscles is moderate to substantial for intra-rater reliability (Kappa coefficient = 0.55–0.97) and acceptable for inter-rater reliability (Kappa coefficient = 0.41–0.54).

Walking speed was subsequently evaluated using the 10 m walk test (10 MWT) [35]. For this test, participants were instructed to walk a 10 m distance at a comfortable speed, without using any walking aids (e.g., walker, cane), although foot orthoses were allowed. The six central meters were timed, allowing two meters at the beginning and at the end of the walkway for acceleration and deceleration, respectively. The test was repeated twice, and the shortest time was used to calculate the walking speed (the distance covered divided by the time taken). In chronic stroke survivors, this test demonstrated excellent test–retest (ICC: 0.85–0.97), intra-rater (ICC: 0.92–0.94), and inter-rater (ICC: 0.96–0.97) reliabilities [35]. Walking speed has been shown to reliably predict an individual’s ability to ambulate in the community [17]. Thus, for statistical purposes, participants were categorized as having (speed < 0.8 m/s) or not having (speed ≥ 0.8 m/s) limited community ambulation.

Finally, BMD was assessed using calcaneal ultrasound densitometry. These measurements were performed using the SONOST 3000 bone densitometer (Osteosys, Seoul, Republic of Korea). SONOST 3000 is a non-invasive, easy-to-use, compact, and portable device commonly used in clinical settings for bone density screening and assessment [36,37]. This method allows us to study the evolution of bone loss and its likelihood of fracture. Calcaneal quantitative ultrasound measurements are generally reliable and reproducible. The short-term coefficients of variation for key parameters, such as speed of sound (SOS), broadband ultrasound attenuation (BUA), and bone quality index (BQI), typically range from 1 to 1.5% for phantom measurements and 1–4% for in vivo measurements [38,39,40,41]. Despite the potential impact of inter-operator and inter-device variability on reproducibility, the implementation of standardized protocols and calibration procedures substantially enhances consistency. Long-term precision remains within acceptable limits for both screening and epidemiological applications, with SOS and BUA coefficients of variation remaining stable over several months [39,41]. Proper positioning and operator technique continue to represent significant sources of measurement error; however, calibration improvements have effectively mitigated these issues. Overall, calcaneal quantitative ultrasound systems provide reliable and reproducible measurements suitable for osteoporosis screening and risk stratification, although their precision remains inferior to that of central DXA for longitudinal monitoring and clinical diagnosis [38,40,41].

The calcaneus of both the right and left heels was measured on a single occasion. This bone is very accessible and convenient to examine and is an excellent reflection of bone metabolic changes, as it is largely composed of trabecular bone [42]. Following the manufacturer’s instructions [43], measurements were performed in a temperature- and humidity-controlled room. Prior to use, the device was calibrated and precision (the manufacturer claims a coefficient of variation of <1%) was measured to reduce the margin of error and ensure accuracy. For calibration purposes [43], a daily test was immediately performed after turning on the device before it got warm. The phantom use for calibration was kept right next to the device when starting it. Then, ultrasound transmission gel was applied to the area of the phantom that was in contact with both probes while the phantom was on the footrest. The amount of the gel applied equaled the area of the phantom in contact with the probe. The phantom was then placed on the footrest, in the device’s measuring position. Next, the daily test was selected from the software menu and the temperature corresponding to the thermo label on the phantom’s surface was then entered. The test was then executed.

For measuring each participant, after data on age, height, weight, ethnicity, and foot size were entered into the device, the inside and outside of the participant’s heel was cleaned with an alcohol wipe, and standard ultrasound gel was applied on them. The heel was then placed on the designated support of the device. During the measurement, participants were instructed to sit still while the densitometer, through two heads located on the inner and outer sides of the heel, generated a series of mechanical vibrations that passed through the calcaneus. The measurements provided were SOS, BUA, BQI, T-score, and Z-score. Table 1 lists all definitions and units of measure of the densitometer parameters.

### 2.4. Statistical Analysis

Because not all the data were normally distributed (Shapiro–Wilk test), the non-parametric analyses were preferred compared with the parametric ones. Data are reported in terms of median (25th–75th percentiles) or relative percentage. Comparisons among groups were performed using the Kruskal–Wallis analysis, followed by the employment of the Mann–Whitney U-test for post hoc pair-wise comparisons. Within-group comparisons (non-affected versus affected limb in the stroke group or dominant versus non-dominant limb in the CG) were studied using the Wilcoxon rank test. The Spearman coefficient (ρ) was used to assess the correlations of different variables with the walking speed. Stepwise multiple linear regression analysis was conducted to identify the factors that were independently associated with the walking speed of participants with stroke. A statistical significance level of 5% was set for all analyses, and the Bonferroni correction was used as appropriate. Analyses were conducted using SPSS Statistics version 28.0.1.1 (IBM, Armonk, NY, USA).

## 3. Results

**Figure 1:**
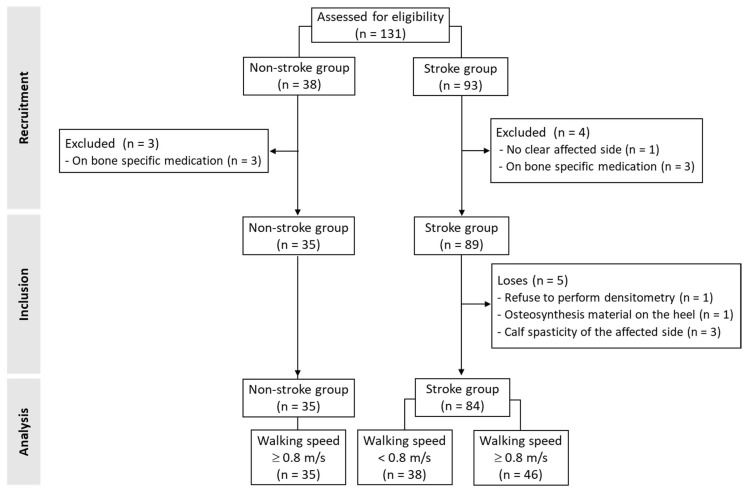
Flow of study participants.

A total of 131 participants (n = 93 with stroke) were recruited (Figure 1). Of them, four subjects with stroke (no clear side affection in medical records [n = 1] and receiving medication for osteoporosis [n = 3]) and three healthy people (all receiving medication for osteoporosis) were excluded. In the stroke group, five subjects were excluded from the analysis due to the unavailability of densitometry data. In this sense, one individual declined to perform the densitometry assessment, another had osteosynthesis material on the affected heel, and three participants had calf spasticity, which prevented the affected heel from resting on the densitometer platform. Finally, data from 84 people with stroke and 35 age- and sex-matched healthy subjects were analyzed.

In the stroke group, average time since stroke was 67 ± 54 months, 54 participants suffered an ischemic stroke (64%), and 52 subjects presented left hemiparesis (62%). Table 2 and Table 3 show the participants’ characteristics when the stroke group was divided by walking speed. Among the sociodemographic variables (Table 2), there were statistically significant differences among groups in height (*p* = 0.049), BMI (*p* < 0.001), alcohol consumption (*p* < 0.001), educational level (*p* < 0.001), and incomes (*p* = 0.002). Participants in the SG were shorter than those in the CG (*p* = 0.017). Stroke participants in both groups (FG and SG) showed higher BMI than those in the CG (*p* = 0.001 and *p* < 0.001, respectively). The proportion of healthy controls who showed a pattern of risk of alcohol consumption was higher than that of stroke participants in either group (*p* = 0.003 for CG versus FG and *p* < 0.001 for CG versus SG). A higher proportion of healthy controls had a higher educational level when compared with the stroke participants in either group (*p* < 0.001 for CG versus FG and *p* = 0.005 for CG versus SG). The FG contained a higher proportion of individuals from lower income brackets when compared with controls (*p* = 0.001). All of them were Caucasian.

The analysis of the clinical variables (Table 3) revealed statistically significant differences among groups in walking speed, disability, ambulation ability, cognitive status, tone of the affected tibialis anterior and gastrocnemius muscles, number of falls, and use of mobility aids (*p* < 0.001 for all the variables). The results obtained by the participants in the SG were inferior to those obtained by the participants in the FG, and both groups demonstrated lower results than the CG in terms of walking speed, disability, and ambulation ability (*p* < 0.001 for all the pair-wise comparisons in all the variables). Stroke participants in both groups (FG and SG) had greater cognitive impairment than those in the CG (*p* = 0.001 and *p* < 0.001, respectively). A higher tone was observed in the tibialis anterior and in the gastrocnemius of the affected leg among the stroke participants (both groups) when compared with the CG (*p* = 0.003 and *p* < 0.001 for FG versus CG, respectively, and *p* < 0.001 for SG versus CG in both muscle groups). For the affected gastrocnemius, participants in the SG demonstrated a higher tone in comparison with those in the FG (*p* < 0.001). Among participants with stroke, a higher number of wheelchair users for outdoor long distances were found in the SG compared with the FG (*p* = 0.020).

With regard to the pharmaceuticals consumed by the participants in the CG, a total of five participants were on drugs that could affect BMD (proton pump inhibitors, n = 2 [2.8%], and levothyroxine, n = 3 [8.5%]). Consequently, Table 3 presents the percentage of participants with stroke on medications that could affect BMD compared by group (FG versus CG).

Table 4 shows the results of the between-group and the between-limbs analyses. In these analyses, the BMD parameters were compared between the affected and non-affected limbs of the same patient or between the non-dominant and dominant limbs of non-stroke subjects. The CG did not show any statistically significant differences between the limbs. The FG showed quite symmetric results; only the BUA value differed significantly between the two limbs (*p* = 0.003), being lower for the affected side. Conversely, all the parameters differed significantly between the two limbs in the SG, with the affected side achieving the worst results. Significant differences were observed between groups for all parameters of the affected limb of participants in the SG, which had lower values than the corresponding limb of both the FG and the CG (SOS: *H* = 13.899, *p* < 0.001; BUA: *H* = 21.363, *p* < 0.001; BQI: *H* = 17.959, *p* < 0.001; T-score: *H* = 17.848; *p* < 0.001; Z-score: *H* = 14.951; *p* < 0.001). However, it is interesting to note that differences were observed for the non-affected limb of participants in the FG, which had higher values than the non-affected limb of participants in the SG in terms of the SOS (*H* = 7.583; *p* = 0.023).

Table 5 reports the correlation analysis in the three groups of subjects. For the CG, speed was found to be significantly correlated only with age. In FG, speed was correlated with height and weight, with functional independence (mRS), ambulation ability (FACHS) and cognitive status (MoCA) among clinical variables, with all the BMD parameters registered in the affected limb and also the SOS in non-affected limb. For SG, a correlation with speed was found only for functional independence (mRS) and ambulation ability (FACHS).

Table 6 reports the results of the linear regression analysis. Four variables that were entered into the model strictly correlated with the measured gait speed (R^2^ = 0.719): the ambulation ability (FACHS), the tone of the affected gastrocnemius muscle (MAS), functional independence (mRS), and the SOS measured in the affected limb. The Variance Inflation Factor (VIF) resulted low values, according to a moderate collinearity of each one of the variables entered into the model with the others. 

## 4. Discussion

In this study, we investigated between-limb differences in BMD of individuals with chronic stroke. We compared those with limited community ambulation with those with non-restricted community ambulation, and we compared both groups with healthy controls. Furthermore, an analysis was conducted to examine the relationship between gait speed and BMD. The results of the study demonstrated that subjects with chronic stroke and full community ambulation speed did not show significant between-limb differences in BMD parameters, with the exception of the BUA, which exhibited a lower value in the affected limb. In contrast, all BMD parameters showed a more pronounced reduction in the affected limb than in the non-affected limb among individuals with a slower walking speed. Furthermore, we demonstrated that SOS was associated with walking speed, among other variables.

Stroke has been previously associated with changes in BMD in the upper and lower affected limbs [8]. In fact, it has been reported that the mean BMD score on the affected side is 7.36% lower than on the non-affected side [46]. However, the observed similarity in BMD parameters between the affected and non-affected limbs of faster post-stroke walkers, with the exception of BUA, suggests a more favorable bone health profile, probably due to their good functional capabilities. The two basic parameters measured by quantitative ultrasound densitometry are SOS and BUA, from which others are derived, such as the Z-score, T-score, and BQI [47]. Overall, SOS and BUA are primarily related to the elasticity, microarchitecture, structural integrity, and porosity of the bone [48], which is, in turn, modulated by the mechanical stress induced by physical activity [49]. Therefore, the BMD results obtained from fast walkers who have had a stroke may be attributed to their higher walking speed, as suggested by previous studies conducted in stroke [50]. This implies that clinicians should prioritize improving gait capacity in post-stroke rehabilitation and highlight the importance of physical activity for this population.

In contrast, all BMD parameters were significantly lower in the affected limb of individuals with slower gait speed after stroke compared with their non-affected limb counterparts. A similar pattern was also observed when comparing the affected limb of slow walkers with the affected limb of faster post-stroke individuals, as well as with the non-dominant leg of healthy controls. These findings imply that individuals with a slow post-stroke gait exhibit a consistent reduction in BMD. This has clinical significance because, for example, lower T-scores may indicate an increased risk of fracture in populations with comorbidities [50], and it can also be used to monitor and set bone treatment goals [51]. This underscores the relevance of assessing BMD in stroke patients to prevent fractures and their associated complications. The Z-score, on the other hand, is inversely related to secondary causes of osteoporosis, meaning that it can help detect bone loss in young people or those approaching menopause [52,53]. In addition, prior studies have found a link between the SOS parameter and elastic modulus and bone density [54]. Moreover, some histomorphometric studies observed that BUA may reflect aspects of bone microarchitecture, such as trabecular separation and connectivity [55]. All these BMD parameters can be modified through targeted exercise interventions and body movement [56]. Therefore, the BMD results obtained by stroke participants with slower walking speeds may be explained by their greater cognitive, musculoskeletal, and functional sequelae, which likely interfere with their ability to perform physical activities of daily living. This reinforces the idea that a comprehensive clinical and rehabilitative approach to stroke is necessary, and that this approach must include physical and psychological aspects to enable patients to perform activities of daily living as optimally as possible.

Interestingly, we also found that, in their non-affected leg, individuals with stroke who were slow walkers showed lower levels of SOS than the fast-walking group with stroke sequelae. Although this leg was not directly affected by the stroke, bone alterations may occur due to various factors. After a stroke, even the non-affected leg can undergo functional changes due to a reduction in ambulation, accompanied by asymmetric gait patterns [57]. Previous studies have shown that bearing less weight on the affected leg occurs during ambulation in people who have had a stroke, and such studies have related this aspect to BMD loss in the non-affected limb [58]. This, in turn, affects the mechanical load on the bone and may alter SOS, as a decrease in the use of the limb has been shown to influence this parameter [59]. During gait, the non-affected limb takes on a compensatory role by generating greater propulsive forces and a change in step asymmetry [57], which can result in higher mechanical stress and potentially harmful bone loading patterns. Furthermore, it is important to consider that, even if the leg is not directly affected, it has been observed that a stroke can still impact overall motor and postural control, leading to functional changes in the non-affected leg [60]. These findings emphasize the importance of assessing overall bone health rather than just the affected side, especially given the significance of gait recovery for this population. To ensure the most harmonious gait pattern and preserve the bone health of the unaffected limb, clinics must ensure that this extremity is not overlooked in the rehabilitation process.

Achieving adequate walking speed after a stroke is essential, as it is closely linked to independence-related outcomes [61]. We observed that in stroke patients, faster gait speed was negatively correlated with disability (measured by the mRS) and muscle tone of the affected gastrocnemius and positively correlated with the ambulation ability (assessed by the FACHS) and cognitive function (assessed by the MoCA). Research findings on the general stroke population are in line with these results [62,63]. A stroke can result in cognitive, musculoskeletal, and functional consequences, with more severe sequelae potentially influencing the reduction in gait [64]. This highlights the need for holistic assessments and rehabilitation in clinical settings.

Meanwhile, walking speed was negatively correlated with the FACHS in people with stroke and slow walking. In the present study, walking aids were not permitted during the assessment of walking speed, with the exception of foot orthoses. This factor may have exerted an influence on the observed gait speed. The FACHS is a classification system that documents the patient’s walking level, without considering the use of walking aids. Consequently, given the higher rate of walking aid users in the SG group (Table 3), slow walkers may have demonstrated superior gait classification in the FACHS in comparison with the performance of the speed test without technical assistance. This observation may also be explained by the fact that the FG exhibits a positive correlation with the FACHS, indicating that they required less technical assistance and were less dependent on it compared with the SG.

Higher walking speeds also appeared to be related to better bone health in the affected and non-affected legs of stroke fast walker participants. This coincides with the previously mentioned idea that greater mechanical loading contributes to higher bone quality and density [10,24,65]. Nonetheless, we must not overlook the influence that anthropometric and sociodemographic characteristics have on walking speed [66]. In our study, walking speed negatively correlated with weight and height in stroke participants who were fast walkers. Higher body weight is associated with slower walking speed because more mass needs to be moved [67]. The negative correlation between height and walking speed contradicts existing literature, which generally suggests that taller people walk faster [68]. However, this relationship may be affected by kinematic factors, such as cadence [69], which were not considered in this study. In addition, age influences walking speed, as age-related physiological changes contribute to its decline [68]. This is consistent with the negative correlation observed in our CG.

Lastly, we found that the FACHS, the mRS, the increased tone of the gastrocnemius muscle in the affected limb, and the BMD-SOS parameter were factors that independently influence the participants’ gait speed, explaining 71.9% of its variance. Although the predictive value of the mRS for walking speed is well established [70], as far as we know, the FACHS scale has not yet been recognized as a predictor of gait speed. Both FACHS and mRS are widely used tools in chronic stroke patients to analyze ambulation ability and functional independence, respectively. Therefore, they can serve as useful predictors of walking speed. Increased tone of the gastrocnemius in the affected limb also had an important effect on gait velocity. Spasticity in the plantar flexors restricts some phases of the gait cycle, such as the propulsion and swing phases, impacting walking speed [71]. Therefore, the most interesting finding was that the SOS measured in the affected limb was also included in the model as a variable that predicts walking speed. Although this finding may seem unexpected at first, it is important to consider that good bone health is necessary for achieving an adequate gait speed. This is particularly true if we take into account the muscle–bone unit theory, which postulates that muscle strength and bone properties form a functional biological unit [72]. Muscle mass imposes mechanical loading on the bones, which stimulates cells to adapt and remodel in response to this stimulus [73]. Thus, this mechanical stress is necessary for bone formation and remodeling, thereby increasing bone density and strength [74]. Without appropriate contraction due to neurological damage and lower physical activity, this process may be altered, and, therefore, the BMD may decrease [9]. Ultimately, lower BMD affects the kinematics of walking, which, consequently, impacts walking speed [75]. This underscores the importance of appropriately evaluating the bones of the stroke population in clinics, since this evaluation may reveal problems that will further affect gait recovery.

### Limitations and Future Work

This study has limitations that should be acknowledged. The results can only be generalized to populations with chronic stroke who are able to walk, with or without a walking aid, as these aspects were part of our selection criteria. In addition, we did not gather information on the participants’ physical activity levels. Therefore, we could not establish a clear relationship between functional limitations and BMD results in relation to physical activity levels. Future studies would benefit from tracking the physical activity of chronic stroke patients to better understand their BMD in relation to their functional capabilities. We assessed BMD using quantitative ultrasound densitometry, which is less precise than dual-energy X-ray absorptiometry. However, ionizing radiation was avoided, and we used the calcaneus, which is a validated measurement [76]. Furthermore, future research could analyze the potential impact of BMD on kinematics to identify potential rehabilitation targets.

## 5. Conclusions

This study reports BMD data of people with chronic stroke depending on their walking speed and compared with a healthy CG. Our results indicate that individuals who had experienced a stroke and had a higher walking speed showed alterations only in the BMD BUA parameter of their affected leg compared with their non-affected leg, reflecting preserved bone health. However, stroke patients with slower walking speeds showed a reduction in all analyzed BMD parameters in their affected leg compared with their non-affected leg. Similarly, all the BMD parameters were lower in this group of participants when compared with the corresponding leg of stroke survivors with a higher walking speed and healthy individuals. This could be attributed to their lower functionality and greater sequelae. Moreover, it is also important to consider both hemibodies in BMD assessment, as a decrease in the SOS parameter examined was observed in the non-affected leg of participants with stroke and slow walking speed. Additionally, a relationship was observed between walking speed and various functionality (muscle tone and cognition) and BMD-related variables. Finally, it is important to emphasize the significance of these factors in determining walking speed in stroke patients, with a particular focus on the predictive value of the SOS of the affected limb. These findings underscore the importance of therapists taking a comprehensive approach to all stroke sequelae and not overlooking the unaffected hemibody during rehabilitation. Our results also highlight the need for therapists to design screening and long-term care strategies that promote bone health among stroke survivors.

## Figures and Tables

**Table 1:** Variables provided by the SONOST 3000 bone densitometer.

Variable	Description	Units
Speed of sound (SOS)	Speed at which the ultrasound signal travels from one transducer to the other	m/s
Broadband ultrasound attenuation (BUA)	Attenuation of the sound waves as they travel from the transmitting transducer to the receiving transducer	dB/MHz
Bone quality index (BQI)	Derived from SOS and BUA with temperature correction and a lower standard deviation value. SONOST 3000 calculates BQI as follows: BQI = αSOS + βBUA (αβ: temperature correction)	%
T-score	Number of standard deviations above or below the mean peak bone mass of the general 30-year-old population matched for sex and ethnic group	Arbitrary units
Z-score	Number of standard deviations above or below the mean bone mass of the population matched for age, sex, and ethnic group	Arbitrary units

**Table 2:** Demographics characteristics among the Fast walking stroke group, the Slow walking stroke group and the control group. Univariate analysis.

	Control Group (n = 35)	Stroke Group (n = 84)		Differences Among Groups
		Fast Group, Walking Speed ≥0.8 m/s (n = 46)	Slow Group, Walking Speed <0.8 m/s (n = 38)	
**Sociodemographic Variables**				
Sex, n (%):				χ^2^ = 0.483; *p* = 0.786
Females	14 (40)	15 (32.6)	14 (36.8)	
Males	21 (60)	31 (67.4)	24 (63.2)	
Age, years	60.00 (53.00–69.00)	54.50 (47.00–64.00)	62.00 (54.00–67.25)	H = 5.304; *p* = 0.070
Stature, m	1.69 (1.65–1.74)	1.67 (66.33–90.40)	1.64 (1.59–1.71)	H = 6.044; ***p* = 0.049**
Weight, kg	71.80 (58.50–79.50)	78.05 (58.50–79.50)	77.20 (67.00–85.40)	H = 4.510; *p* = 0.105
Body mass index, kg/m^2^	24.99 (21.75–27.06)	28.33 (24.50–31.04)	28.34 (24.74–32.04)	H = 13.955; ***p* < 0.001** †‡
Smoking, n (%):				χ^2^ = 2.542; *p* = 0.649
Non-smoker	15 (42.9)	14 (30.4)	11 (28.9)	
Ex-smoker	17 (48.6)	26 (56.5)	24 (63.2)	
Smoker	3 (8.6)	6 (13.0)	3 (7.9)	
Alcohol consumption habits, n (%):	n = 35	n = 46	n = 37	χ^2^ = 17.944; ***p* < 0.001** †‡
Zero or low-risk	18 (51.4)	38 (82.6)	34 (91.9)	
At risk *	17 (48.6)	8 (17.4)	3 (8.1)	
Educational level, n (%):	n = 35	n = 46	n = 37	χ^2^ = 24.632; ***p* < 0.001** †‡
None	0 (0.0)	2 (4.3)	1 (2.7)	
Primary	3 (8.6)	17 (37.0)	9 (24.3)	
Secondary	4 (11.4)	15 (32.6)	12 (32.4)	
University	28 (80.0)	12 (26.1)	15 (40.5)	
Incomes, n (%):	n = 35	n = 45	n = 37	χ^2^ = 16.154; ***p* = 0.002** †‡
Low	1 (2.9)	10 (22.2)	3 (8.1)	
Medium	17 (48.6)	28 (62.2)	27 (73.0)	
High	17 (48.6)	7 (15.6)	7 (18.9)	

Data are shown as median (25th–75th percentiles) unless otherwise specified. The last column reports the *p*-values of the among-group differences analysis (Kruskal–Wallis’ test or Chi-Square test, as appropriate). Significance for post hoc pair-wise comparisons (Mann–Whitney U test or Chi-Square test, as appropriate) was adjusted by Bonferroni correction *(p* < 0.0167) [44] and is reported as follows: † the stroke group with unlimited community ambulation (fast group) versus the control group; and ‡ the stroke group with limited community ambulation (slow group) versus control group. * A pattern of risky alcohol consumption was defined as men drinking > 300 mL/day and women drinking > 208 mL/day [45]. Statistically significant differences are highlighted in bold.

**Table 3:** Clinical characteristics among the Fast walking stroke group, the Slow walking stroke group and the control group. Univariate analysis.

	Control Group (n = 35)	Stroke Group (n = 84)		Differences Among Groups
		Fast Group, Walking Speed ≥0.8 m/s (n = 46)	Slow Group, Walking Speed <0.8 m/s (n = 38)	
**Clinical Variables**				
Walking speed, 10 MWT, m/s	1.39 (1.30–1.47)	0.99 (0.86–1.27)	0.47 (0.34–0.63)	H = 14.931; ***p* < 0.001** †‡§
Functional independence, mRS	0.00 (0.00–0.00)	2.00 (1.00–2.00)	3.00 (2.00–4.00)	H = 85.412; ***p* < 0.001** †‡§
Ambulation ability, FACHS	5.00 (5.00–5.00)	4.00 (4.00–5.00)	3.00 (2.00–3.25)	H = 82.877; ***p* < 0.001** †‡§
Cognitive status, MOCA	27.00 (25.00–29.00)	23.50 (19.00–26.00)	23.00 (20.50–25.00)	H = 32.142; ***p* < 0.001** †‡
Muscle spasticity, MAS				
Tibialis anterior AF	0.00 (0.00–0.00)	0.00 (0.00–0.00)	0.00 (0.00–2.00)	H = 19.410; ***p* < 0.001** †‡
Tibialis anterior NA	0.00 (0.00–0.00)	0.00 (0.00–0.00)	0.00 (0.00–0.00)	H = 0.000; *p* = 1.000
Gastrocnemius AF	0.00 (0.00–0.00)	0.50 (0.00–2.00)	3.00 (1.00–4.00)	H = 53.102; ***p* < 0.001** †‡§
Gastrocnemius NA	0.00 (0.00–0.00)	0.00 (0.00–0.00)	0.00 (0.00–0.00)	H = 1.587; *p* = 0.452
Number of falls	0.00 (0.00–0.00)	0.00 (0.00–1.00)	0.50 (0.00–1.25)	H = 14.931; ***p* < 0.001** †‡
Time since stroke, months	-	50.00 (27.50–87.25)	55.00 (27.75–95.00)	U = 814.500; *p* = 0.593
Type of stroke, n (%):				χ^2^ = 1.234; *p* = 0.267
Ischemic	-	32 (69.6)	22 (57.9)	
Hemorrhagic		14 (30.4)	16 (42.1)	
Medication, n (%):				
SSRI	-	15 (32.6)	15 (39.5)	χ^2^ = 0.427; *p* = 0.513
Neuroleptics	-	4 (8.7)	2 (5.3)	χ^2^ = 0.370; *p* = 0.685
Corticoids	-	2 (4.3)	1 (2.6)	χ^2^ = 0.178; *p* = 1.000
Heparin	-	1 (2.2)	1 (2.6)	χ^2^ = 0.019; *p* = 1.000
PPIs	-	22 (47.8)	18 47.4)	χ^2^ = 0.002; *p* = 0.967
Antiepileptics	-	10 (21.7)	10 (26.3)	χ^2^ = 0.240; *p* = 0.624
Chemotherapy	-	0 (0.0)	1 (2.6)	χ^2^ = 1.225; *p* = 0.452
Levothyroxine	-	1 (2.2)	2 (5.3)	χ^2^ = 0.577; *p* = 0.587
Use of mobility aids, n (%):	-	14 (30.4)	33 (86.8)	χ^2^ = 26.865; ***p* < 0.001**
Wheelchair	-	0 (0.0)	11 (33.3)	χ^2^ = 6.093; ***p* = 0.020**
Cane	-	8 (51.7)	22 (66.7)	χ^2^ = 0.386; *p* = 0.534
Tripod cane	-	0 (0.0)	5 (15.2)	χ^2^ = 2.374; *p* = 0.303
Frame	-	0 (0.0)	1 (3.0)	χ^2^ = 0.433; *p* = 1.000
Foot up splint		8 (57.1)	16 (48.5)	χ^2^ = 0.295; *p* = 0.587

Data are shown as median (25th–75th percentiles) unless otherwise specified. The last column reports the *p*-values of the among-group differences analysis (Kruskal–Wallis’ test, Chi-Square test, or Mann–Whitney U test, as appropriate). Significance for post hoc pair-wise comparisons (Mann–Whitney U test or Chi-Square test, as appropriate) was adjusted by Bonferroni correction (*p* < 0.0167) [44] and is reported as follows: § stroke group with unlimited community ambulation (fast group) versus the stroke group with limited community ambulation (slow group); † fast group versus control group; and ‡ slow group versus control group. Statistically significant differences are highlighted in bold. 10 MWT: 10 m walk test, mRS: modified Rankin scale, FACHS: Functional Ambulation Classification of the Hospital of Sagunto, MOCA: Montreal cognitive assessment, MAS: Modified Ashworth scale, AF: Affected limb, NA: Non-affected limb, SSRI: Selective serotonin reuptake inhibitors, PPIs: Proton pump inhibitors.

**Table 4:** Comparison of bone mineral density parameters among the Fast walking stroke group, the Slow walking stroke group and the control group, and between limbs within each group. Univariate analysis.

		CG (n = 35)	Stroke Group (n = 84)		Between-Group Analysis		
			FG (n = 46)	SG (n = 38)	CG vs. FG	CG vs. SG	FG vs. SG
**SOS**	AF/ND limb	1531.58 (1521.03–1539.40)	1523.56 (1516.41–1542.47)	1513.32 (1501.23–1530.72)	*U* = 732.000; *p* = 0.486; *r* = 0.077	*U* = 347.000; ***p* < 0.001**; *r* = 0.411	*U* = 551.000; ***p* = 0.004**; *r* = 0.317
	NA/D limb	1528.00 (1521.09–1539.66)	1531.38 (1516.52–1542.07)	1521.39 (1506.90–1532.26)	*U* = 793.000; *p* = 0.909; *r* = 0.013	*U* = 459.000; *p* = 0.023; *r* = 0.266	*U* = 597.000; ***p* = 0.013**; *r* = 0.272
	Differences between-limbs	Z = −1.261; *p* = 0.207 *r* = 0.213	Z = −0.186; *p* = 0.853; *r* = 0.027	Z = −2.465; ***p* = 0.014**; *r* = 0.400			
**BUA**	AF/ND limb	101.93 (92.10–116.68)	91.71 (83.14–111.15)	82.39 (70.45–94.79)	*U* = 581.000; *p* = 0.033; *r* = 0.237	*U* = 256.000; ***p* < 0.001**; *r* = 0.592	*U* = 559.000; ***p* = 0.005**; *r* = 0.309
	NA/D limb	103.28 (90.44–114.34)	97.40 (87.78–114.39)	92.16 (83.48–105.26)	*U* = 710.500; *p* = 0.368; *r* = 0.100	*U* = 461.500; *p* = 0.025; *r* = 0.263	*U* = 687.000; *p* = 0.093; *r* = 0.183
	Differences between-limbs	Z = −0.229; *p* = 0.819; *r* = 0.039	Z = −2.929; ***p* = 0.003**; *r* = 0.432	Z = −4.300; ***p* < 0.001**; *r* = 0.698			
**BQI**	AF/ND limb	100.55 (87.90–112.65)	93.45 (79.53–112.54)	78.55 (65.72–96.59)	*U* = 662.000; *p* = 0.173; *r* = 0.151	*U* = 290.000; ***p* < 0.001**; *r* = 0.485	*U* = 547.000; ***p* = 0.003**; *r* = 0.321
	NA/D limb	96.71 (85.90–115.75)	99.62 (82.76–111.31)	90.49 (72.00–102.39)	*U* = 766.000; *p* = 0.710; *r* = 0.041	*U* = 454.000; *p* = 0.020; *r* = 0.273	*U* = 611.000; *p* = 0.018; *r* = 0.258
	Differences between-limbs	Z = −0.811; *p* = 0.417; *r* = 0.137	Z = −1.732; *p* = 0.083; *r* = 0.255	Z = −4.020; ***p* < 0.001**; *r* = 0.652			
**T-score**	AF/ND limb	−0.24 (−0.92–0.41)	−0.62 (−1.37–0.41)	−1.42 (−2.12–−0.46)	*U* = 662.500; *p* = 0.174; *r* = 0.151	*U* = 290.500; ***p* < 0.001**; *r* = 0.484	*U* = 549.000; ***p* = 0.003**; *r* = 0.319
	NA/D limb	−0.45 (−1.03–0.58)	−0.29 (−1.20–0.34)	−0.78 (−1.76–−0.14)	*U* = 766.000; *p* = 0.710; *r* = 0.041	*U* = 452.500; *p* = 0.019; *r* = 0.275	*U* = 612.000; *p* = 0.019; *r* = 0.257
	Differences between-limbs	Z = −0.803; *p* = 0.422; *r* = 0.136	Z = −1.750; *p* = 0.080; *r* = 0.258	Z = −4.021; ***p* < 0.001**; *r* = 0.652			
**Z-score**	AF/ND limb	0.35 (−0.52–0.99)	−0.17 (−0.89–0.80)	−0.91 (−1.51–0.27)	*U* = 642.000; *p* = 0.120; *r* = 0.173	*U* = 315.000; ***p* < 0.001**; *r* = 0.452	*U* = 606.500; ***p* = 0.016**; *r* = 0.262
	NA/D limb	0.09 (−0.45–1.37)	0.08 (−0.61–1.04)	−0.42 (−0.90–0.53)	*U* = 756.000; *p* = 0.640; *r* = 0.052	*U* = 478.500; *p* = 0.039; *r* = 0.241	*U* = 709.000; *p* = 0.138; *r* = 0.162
	Differences between-limbs	Z = −0.917; *p* = 0.359; *r* = 0.155	Z = −1.699; *p* = 0.089; *r* = 0.251	Z = −3.915; ***p* < 0.001**; *r* = 0.635			

Data are shown as median (25th–75th percentiles). The last column reports the significance for post hoc pairwise comparisons (Mann–Whitney U test) adjusted by Bonferroni correction (*p* < 0.0167 [29]) and the effect sizes. The *p*-values of the within-subject comparisons (Wilcoxon test) and the effect sizes are reported in the rows. Statistically significant differences are highlighted in bold. CG: Non-stroke group; FG: Fast group (walking speed ≥ 0.8 m/s); SG: Slow group (walking speed < 0.8 m/s); SOS: Speed of sound; BUA: Broadband ultrasound attenuation; BQI: Bone quality index; AF/ND: Affected/non-dominant; NA/D: Non-affected/dominant.

**Table 5:** Relationship of demographics, clinical and bone mineral density variables with walking speed for the Fast walking stroke group, the Slow walking stroke group and the control group. Bivariate analysis.

Correlation with Walking Speed (ρ Values)	Control Group (n = 35)	Stroke Group (n = 84)	
		Fast Group, Walking Speed ≥0.8 m/s (n = 46)	Slow Group, Walking Speed <0.8 m/s (n = 38)
Age	−0.459 *	−0.107	−0.246
Stature	0.022	**−0.357** *	−0.115
Weight	−0.012	**−0.316** *	−0.279
Body Mass Index	−0.080	−0.207	−0.245
Functional independence, mRS	-	**−0.498** **	**−0.660** **
Ambulation ability, FACHS	-	**0.608** **	**−0.653** **
Cognitive status, MoCA	−0.032	**0.300** *	−0.018
MAS tibialis anterior AF	-	−0.229	−0.160
MAS tibialis anterior NAF	-	0.000	0.000
MAS gastrocnemius AF	-	**−0.391** *	−0.057
MAS gastrocnemius NAF	-	−0.118	0.000
SOS affected/non-dominant limb	0.002	**0.362** *	0.122
BUA affected/non-dominant limb	0.053	**0.348** *	0.146
BQI affected/non-dominant limb	0.034	**0.389** *	0.106
T-score affected/non-dominant limb	0.034	**0.389** *	0.109
Z-score affected/non-dominant limb	0.002	**0.372** *	0.091
SOS non-affected/dominant limb	0.003	**0.298** *	0.214
BUA non-affected/dominant limb	0.135	0.119	0.113
BQI non-affected/dominant limb	0.058	0.267	0.193
T-score non-affected/dominant limb	0.060	0.266	0.195
Z-score non-affected/dominant limb	0.009	0.210	0.142

Statistically significant results are highlighted in bold (* *p* < 0.05 and ** *p* < 0.001). AF: affected limb, NAF: non-affected limb.

**Table 6:** Factors independently associated with the walking speed of participants with stroke. Results of the multiple linear regression analysis.

	B-Coefficient (95% Interval of Confidence)	Beta-Coefficient	*p*-Value	Collinearity VIF
Ambulation ability, FACHS	0.21 (−0.76; −0.03)	0.531	<0.001	2.326
Gastrocnemius mAS-AF	−0.05 (−0.08; −0.02)	−0.213	0.001	1.159
Functional independence, mRS	−0.072 (−0.14; −0.01)	−0.195	0.027	2.095
SOS-AF	0.003 (0.001; 0.01)	0.138	0.035	1.160

The parameters of the prediction model resulted: F(4,839) = 50.415, *p* < 0.001, R^2^ = 0.719, adjusted R^2^ = 0.704. FACHS: Functional Ambulation Classification of the Hospital of Sagunto; mRS: modified Rankin Scale; mAS: Modified Ashworth Scale; AF: affected limb; SOS: speed of sound; VIF: Variance Inflation Factor.

## Data Availability

The data that support the findings of this study are available from the corresponding author, [M.L.S.-S.], upon reasonable request.

## Supplementary Materials

The following supporting information can be downloaded at: https://www.mdpi.com/article/10.3390/jcm14238426/s1.

## Abbreviations

The following abbreviations are used in this manuscript:

BMI	Body mass index
BMD	Bone mineral density
BQI	Bone quality index
BUA	Broadband ultrasound attenuation
CG	Control group
FACHS	Functional Ambulation Classification of Hospital of Sagunto
FG	Stroke participants with fast walking speed (FG ≥ 0.8 m/s)
SG	Stroke participants with slow walking speed (SG < 0.8 m/s)
SOS	Speed of sound
